# Following instructions in a dual-task paradigm: Evidence for a temporary motor store in working memory

**DOI:** 10.1177/1747021817743492

**Published:** 2018-01-01

**Authors:** Agnieszka J Jaroslawska, Susan E Gathercole, Joni Holmes

**Affiliations:** 1 School of Philosophy, Psychology & Language Sciences, The University of Edinburgh, Edinburgh, UK; 2 MRC Cognition and Brain Sciences Unit, University of Cambridge, Cambridge, UK

**Keywords:** Working memory, dual-task, following instructions, motor store, multiple component model

## Abstract

Evidence from dual-task studies suggests that working memory supports the retention and implementation of verbal instructions. One key finding that is not readily accommodated by existing models of working memory is that participants are consistently more accurate at physically performing rather than verbally repeating a sequence of commands. This action advantage has no obvious source within the multi-component model of working memory and has been proposed to be driven by an as yet undetected limited-capacity store dedicated to the temporary maintenance of spatial, motoric, and temporal features of intended movements. To test this hypothesis, we sought to selectively disrupt the action advantage with concurrent motor suppression. In three dual-task experiments, young adults’ immediate memory for sequences of spoken instructions was assessed by both action-based and spoken recall. In addition to classic interference tasks known to tax the phonological loop and central executive, motor suppression tasks designed to impair the encoding and retention of motoric representations were included. These required participants to produce repetitive sequences of either fine motor gestures (Experiment 1, *N* = 16) or more basic ones (Experiments 2, *N* = 16, and 3, *N* = 16). The benefit of action-based recall was reduced following the production of basic gestures but remained intact under all other interference conditions. These results suggest that the mnemonic advantage of enacted recall depends on a cognitive system dedicated to the temporary maintenance of motoric representations of planned action sequences.

## Introduction

Recent studies of instruction-guided behaviour have recognised that the capacity to hold in mind the content of the instruction while simultaneously performing each step in turn is supported by working memory—a cognitive system combining limited-capacity storage with attentional control (e.g., Engle, Carullo, & Collins, 1991; Gathercole, Durling, Evans, Jeffcock, & Stone, 2008; Jaroslawska, Gathercole, Logie, & Holmes, 2016; Kim, Bayles, & Beeson, 2008; Yang, Allen, & Gathercole, 2016; Yang, Gathercole, & Allen, 2014). There is also evidence that the recall of instructions can be enhanced by physical movement and that information encoded for the purpose of future action is stored or organised differently from information retained for future verbal recall (e.g., Allen & Waterman, 2015; Gathercole et al., 2008; Jaroslawska, Gathercole, Allen, & Holmes, 2016; Waterman et al., 2017; Yang, Allen, Yu, & Chan, 2015; Yang et al., 2014). A striking example of the difference between memory for to-be-repeated and to-be-performed tasks is the benefit of physical performance over verbal repetition at recall, termed the action advantage (e.g., Allen & Waterman, 2015; Gathercole et al., 2008; Jaroslawska, Gathercole, Allen, & Holmes, 2016; Koriat, Ben-Zur, & Nussbaum, 1990; Waterman et al., 2017; Yang, Allen, Holmes, & Chan, 2017; Yang et al., 2015; Yang et al., 2014). While the action advantage is consistent and robust, relatively little is known about its precise cognitive underpinnings.

In three experiments we investigated evidence suggesting that this effect arises from a dedicated temporary motor store located within working memory. The working memory model of Baddeley and Hitch (1974; Baddeley, 2000, 2003; Baddeley, Allen, & Hitch, 2011) was used to guide these investigations. This model consists of a central executive responsible for attentional control within and beyond working memory that is supported by two specialised limited-capacity stores: the phonological loop and visuospatial sketchpad (Baddeley, 2000, 2003; Baddeley et al., 2011; Baddeley & Hitch, 1974). The phonological loop maintains verbal and acoustic information in a temporary store using an articulatory rehearsal system, while the visuospatial sketchpad maintains nonverbal information and is assumed to be fractionated into separate visual, spatial, and possibly kinaesthetic components (e.g., Logie, 1995, 2011; Smyth & Pendleton, 1989, 1990). A fourth component, the episodic buffer is a limited-capacity store capable of multi-dimensional coding that forms an interface between the subsystems of working memory and long-term memory (Baddeley, 2000). Empirical work distinguishing the different components of working memory has relied on dual-task methodologies. These require participants to undertake a memory task while concurrently performing a secondary task during encoding, maintenance, or retrieval. The underlying assumption of this approach is that when performed simultaneously, tasks that rely on the same component of working memory will compete for cognitive resources, while tasks drawing on different components will not. Consequently, dual-task costs will emerge only when the two tasks tap the same component of working memory.

The dual-task methodology has recently been used to determine how the multi-component model of working memory contributes to instruction following. Seeking to isolate the cognitive source of the action advantage, Yang et al. (2014) gave young adults spoken instructions to perform a series of actions on objects and then asked them to either physically perform or verbally recall the sequence. Participants were also required to engage in concurrent articulatory suppression, backward counting, and spatial tapping during the encoding of instructions to disrupt the phonological loop, central executive, and visuospatial sketchpad components of working memory, respectively. Recall accuracy was substantially disrupted by all three concurrent activities, indicating that the encoding and retention of verbal instructions depends on multiple aspects of working memory. Crucially, the action advantage remained intact under all three dual-task conditions and enacted recall was always more accurate than verbal recall. This finding was subsequently replicated using spoken rather than written instruction sequences (Yang et al., 2016).

The apparent resistance of the action advantage to concurrent interference has two implications. First, it suggests that the benefit of action over verbal repetition has no obvious source within the existing multi-component framework of working memory. Second, it indicates that storing instructions for subsequent physical implementation involves factors additional to those involved in simple verbal repetition. Consistent with this, Koriat et al. (1990) suggested that the recall of short sequences of action phrases relating to real objects (e.g., *move the eraser, lift the cup*) was improved on a surprise verbal test (when participants were anticipating action recall) due to the benefits of action planning during encoding. That is, action commands were encoded in a motoric form to take advantage of the richness of the visual and kinaesthetic representations that underlie action performance, and these benefitted subsequent verbal recall. This interpretation was recently echoed by Allen and Waterman (2015) who argued that participants make use of spatial–motoric action representations when anticipating enactment at recall, and that these supplement the phonological code generated by verbal instructions leading to improved memory performance.

What system might be responsible for the encoding and temporary storage of spatial–motoric representations? One possibility is that the visuospatial sketchpad component of working memory is fractionated beyond visual and spatial subsystems (see Klauer & Zhao, 2004, for review). For example, Baddeley (2012) noted that many types of motor movement have been shown to interfere with storage in visuospatial working memory. These include spatial tapping of keys on a three-dimensional (3D) display (e.g., Logie & Marchetti, 1991), pursuit tracking of a small circle as it moves around a computer screen (e.g., Baddeley & Lieberman, 1980), arm movements across an unseen matrix (e.g., Quinn, 1994), and eye movements (e.g., Pearson & Sahraie, 2003; Postle, 2006). However, these movements are all target-oriented—they are directed towards specific targets in space that have precise spatial coordinates and necessarily require visuospatial processing. It has been speculated that configural movements (i.e., movements that do not entail the encoding of a target location in external space; for example, performing an arabesque) rely less on visuospatial processing and do not therefore necessarily involve the sketchpad (e.g., Cortese & Rossi-Arnaud, 2010; Smyth & Pendleton, 1989, 1990).

An alternative possibility is that there is an additional slave system within working memory that is dedicated to handling patterns of body movements (Smyth & Pendleton, 1989, 1990). This is supported by a double dissociation between tasks involving moving to targets in external space and movements focused purely on the configurations of body parts (Smyth, Pearson, & Pendleton, 1988). Smyth and Pendleton (1989) found that spatial tapping interfered with memory for a sequence of spatial locations, whereas hand clenching had a detrimental effect on memory for gestures and body configurations. Similarly, recall of a sequence of configural movements was disrupted by concurrently performing, watching, or encoding patterned movements but not by simultaneously encoding a sequence of spatial positions (Smyth & Pendleton, 1990). This dissociation is further supported by a study in which climbers were trained on two different routes on a climbing wall: one vertical and one horizontal (Smyth & Waller, 1998). The distance of the climb that could be seen from the start position and the visibility of the holds were both greater in the vertical climb, but the need to use a variety of hand and body configurations was greater in the horizontal climb. After physical training that involved completing both climbs, participants were required to imagine climbing the routes under dual-task conditions. The secondary tasks were as follows: dynamic visual noise, spatial tapping, and kinaesthetic suppression (i.e., hand clenching). Smyth and Waller (1998) found that the vertical route, designed to emphasise visual resources in the imagery task, took longer to imagine when paired with dynamic visual noise, a task which has obligatory access to visual processing mechanisms (e.g., Quinn & McConnell, 2006). The horizontal route, chosen to highlight kinaesthetic or configural aspects of imagery, took longer to imagine when paired with the hand-clenching task. Imagining both routes was slowed by concurrent spatial tapping, which is unsurprising given that wall climbing is essentially performing a sequence of target-oriented hand movements.

Smyth and Pendleton (1989, 1990) proposed that the motor store is involved in reproducing the configural aspects of physical movement, whereas the visuospatial sketchpad (as it is currently conceptualised) is spatial in character. The motor store hypothesis fits well with recent findings from Shebani and Pulvermüller (2013) who reported that rhythmic movements of either the hands or the feet led to a differential impairment in working memory for concurrent arm- and leg-related action words, with hand and arm movements predominantly impairing working memory for words used to describe arm actions and foot or leg movements primarily impairing leg-related words. Using the rationale behind the dual-task methodology, Shebani and Pulvermüller (2013) concluded that body movements and working memory for action-related words share processing resources.

The primary objective of the current set of experiments is to pinpoint the source of the action advantage during the recall of instructions and to test the hypothesis that the spatial, motoric, and temporal features of planned actions are encoded in a temporary motor store within working memory (Jaroslawska, Gathercole, Allen, & Holmes, 2016; Smyth & Pendleton, 1989, 1990). Representations in this store are generated either by physical performance or by planning to enact and provide an additional source of mnemonic information to supplement verbal and visuospatial storage in working memory. It is beyond the scope of this report to determine whether the hypothesised motor store is a distinct slave system within the multi-component framework or a fractionated subsystem of the visuospatial sketchpad.

Experiment 1 tested the motor store hypothesis using a dual-task methodology to isolate components of working memory involved in following instructions. In addition to concurrent tasks used to disrupt the phonological loop and central executive, a motor suppression task was included. This was designed to impair the encoding and retention of motoric representations and involved the repetitive production of short sequences of complex configural motor gestures adapted from Smyth and Pendleton (1989, 1990). The impact of these concurrent activities was tested on both verbal and action-based recall. Based on previous findings from Yang et al. (2016) and Yang et al. (2014), it was predicted that there would be a substantial benefit of action-based recall over verbal repetition in the baseline condition (no interference) and under conditions designed to disrupt the existing subsystems of the multi-component model of working memory. In line with the motor store hypothesis, motor suppression was predicted to selectively diminish the action advantage at recall by disrupting the motoric encoding of planned action sequences.

## Experiment 1

### Method

#### Participants

In total, 16 right-handed adults (12 females) with a mean age of 23.13 years (standard deviation [*SD*] = 3.65) ranging between 18 and 29 years took part in this experiment. This sample size was estimated to provide power >.95 for effect sizes >.35 for the main analysis. All volunteers were native English speakers, had normal or corrected-to-normal vision, and no history of neurological disorders. Participants were recruited via the MRC Cognition and Brain Sciences Unit volunteer panel using an online booking system and received a small honorarium in return for taking part in the study.

#### Materials

##### Primary task

The following instructions paradigm developed by Gathercole et al. (2008), in which participants were required to carry out sequences of instructions on an array of props laid out in front of them, was adapted for this study. The instruction sequences consisted of descriptions of actions to be performed on a set of concrete, 3D props. The objects were a set of five stationery items (a ruler, an eraser, a pencil, a folder, and a box), in each of three colours (red, yellow, or blue). There were two actions: touch (e.g., *Touch the red pencil*) and pick up (e.g., *Pick up the yellow ruler*). Actions involving touching and picking up were concatenated using the adverb “then” in order to produce increasingly longer sequences that varied in length but not in lexical complexity. The items used in each instruction were selected at random, with the constraint that there was no repetition of colour and object combination in the instruction as a whole. A fixed sequence length of four object–action pairs (e.g., *Pick up the yellow folder, then touch the blue pencil, then touch the red ruler, and then pick up the blue box*) was used in this study and in Experiments 2 and 3. Pilot work established this as the optimal length to avoid floor and ceiling effects. There were 12 trials within each experimental block (two practice trials and 10 test trials). The practice trials were not recorded or analysed. Responses obtained in the experimental trials were recorded by the experimenter in real time and scored as correct if the action–colour–object combinations were recalled in their correct serial position in the sequence. Performance was reported in terms of the proportion of action phrases recalled correctly, out of possible 40 per block (i.e., four action–object pairs per trial, across 10 trials).

##### Secondary tasks

There were three concurrent interference tasks: articulatory suppression, backward counting, and motor suppression. In the articulatory suppression condition, participants were instructed to continuously recite *one, two, three* to prevent verbal rehearsal and block the phonological loop (Baddeley, Thomson, & Buchanan, 1975). Backward counting required continuous deduction of three from a given three-digit number (e.g., *679, 676, 673*) and imposed demands on both the central executive and phonological loop. The number from which participants had to begin the subtraction was different on each trial (Allen, Baddeley, & Hitch, 2006; Baddeley, Hitch, & Allen, 2009; Postma & De Haan, 1996). In the motor suppression condition, participants were instructed to produce a short repetitive sequence of fine motor gestures with their dominant (i.e., right) hand. A set of three hand movements was chosen and adapted from Smyth and Pendleton (1989) and included the following: an open palm (fingers spread apart, hand palm down, fingertips away from body, wrist straight), a fist (fingers curled tightly into a fist, thumb tucked in, wrist straight), and a pointing finger (index finger pointing forwards, remaining fingers curled into a fist, wrist straight). The secondary tasks were self-paced. Participants were instructed to perform these continuously and consistently throughout and to be as fast and accurate as possible.

#### Design and procedure

The experiment implemented a 2 × 4 repeated measures design manipulating recall format (verbal, action) and concurrent interference (no interference, articulatory suppression, backward counting, motor suppression) with each of the eight resulting conditions performed in a separate block. Condition order was counterbalanced across participants, with all those of a particular recall format performed together. The order of the interference conditions was randomised between participants.

Participants were invited to the MRC Cognition and Brain Sciences Unit and assessed individually on eight conditions of the following instructions task. Testing was completed during a single session lasting approximately 60 min. At the beginning of each session, the participants were familiarised with the objects and their labels and with what each physical action involved. Following that, the secondary tasks were practised in isolation until their performance was fluent and error free. On each trial, a concurrent task was initiated 5 s before the presentation of instructions and ceased at the recall cue. A period of 1 s was interpolated between the end of the instructions and the recall cue. Instruction sequences were read aloud by the experimenter at a rate of approximately 3 s per individual action phrase and experimental props remained in sight at all times. At recall, participants either physically enacted or verbally repeated the instructions. Recall was self-paced in all conditions.

Written consent was obtained prior to testing. The study was approved and conducted in accordance with the guidelines of the Cambridge University Psychology Research Ethics Committee and the MRC Cognition and Brain Sciences Unit (Ethical Approval Pre.2014.87). These ethical requirements were also met for Experiments 2 and 3.

### Results

**Figure 1.**
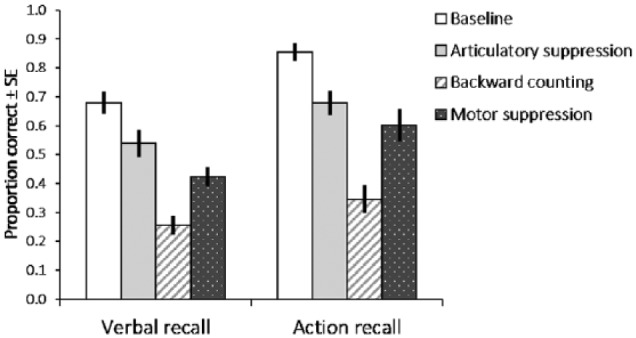
The effects of concurrent articulatory suppression, backward counting, and motor suppression on performance on the following instructions task as a function of two types of recall (verbal or enacted) in Experiment 1. Error bars denote standard error.

The dependent variable was the proportion of action–object pairs correctly recalled in each condition (summarised in Figure 1). The data revealed two patterns that are of interest here. First, across all experimental conditions, action-based recall was more accurate than verbal recall. Second, instruction following was impaired by all three secondary tasks, irrespective of the type of recall required at test.

A 2 × 4 repeated measures ANOVA with factors of recall (verbal, action) and concurrent task (no interference, articulatory suppression, backward counting, motor suppression) revealed a significant main effect of recall type, *F*(1, 15) = 27.296, mean squared error [*MSE*] = .025, *p* < .001, $\eta_{p}^{2}=.645$, with enacted recall significantly more accurate than verbal recall. The main effect of concurrent task was also significant, *F*(3, 45) = 62.426, *MSE* = .020, *p* < .001, $\eta_{p}^{2}=.806$. Simple main effects analysis indicated that recall accuracy in all concurrent task conditions (collapsed across both types of recall) was significantly poorer than in the baseline condition (all *p* < .001, Bonferroni-adjusted). The interaction between recall type and concurrent task was not significant, *F*(3, 45) = 1.555, *MSE* = .009, *p* = .213, $\eta_{p}^{2}=.094$. Thus, the secondary tasks had an equivalent effect on performance regardless of whether the instructions were verbally repeated or performed physically at retrieval.

Planned comparisons (paired *t* tests) confirmed that action recall was significantly better than verbal recall across all conditions: baseline, *t*(15) = 5.464, *p* < .001, Cohen’s *d* = 1.37; articulatory suppression, *t*(15) = 3.024, *p* = .004, *d* = .76; backward counting, *t*(15) = 2.781, *p* = .007, *d* = .70; and motor suppression, *t*(15) = 3.783, *p* < .001, *d* = .95.

### Discussion

The aim of this experiment was to investigate whether a motor suppression task involving a sequence of fine motor gestures selectively eliminated the action advantage at recall. There were three key findings. First, enacted recall was superior to verbal recall across all conditions. Second, articulatory suppression and backward counting disrupted performance in both recall conditions. Third, a motor suppression task involving repetitive sequences of hand gestures had a similar deleterious effect on recall accuracy. Like the other suppression tasks, it failed to diminish the action advantage at recall. The benefit for enactment over verbal repetition replicates previous observations of an action advantage (Allen & Waterman, 2015; Gathercole et al., 2008; Jaroslawska, Gathercole, Allen, & Holmes, 2016; Koriat et al., 1990; Yang et al., 2016; Yang et al., 2017; Yang et al., 2014). This effect may arise through increased engagement with additional forms of coding that provide richer and more robust representations for action recall than those serving the verbal recall of instruction sequences.

Irrespective of the recall modality, both articulatory suppression and backward counting impaired memory for instructions. This is consistent with evidence that people with verbal short-term memory deficits struggle to perform a series of actions to verbal instruction (Cohen-Mimran & Sapir, 2007; Smith, Mann, & Shankweiler, 1986). It also underscores a role for the phonological loop in storing the verbal content of spoken or written instructions, with the central executive coordinating the execution of actions through the retrieval of information from the phonological loop (Jaroslawska, Gathercole, Logie, & Holmes, 2016; Yang et al., 2016; Yang et al., 2014). Neither concurrent task reduced the advantage afforded to physical action at recall, replicating Yang et al.’s (Yang et al., 2016; Yang et al., 2014) reports that neither the central executive nor the phonological loop appear to be the source of action advantage. Notably, performance in the backward counting condition was very poor (less than 40% accuracy). These possible floor effects suggest that the demands of the secondary task were too high.

Contrary to expectations, the concurrent motor suppression task failed to abolish the action advantage. While the concurrent continuous production of gestures had a negative impact on performance in the action recall condition, it also impacted on spoken repetition. This may be because that there is no dedicated motor store in working memory. The benefits of action at recall might instead have deep roots in sensorimotor processing. This interpretation is consistent with an embodied approach to cognition (e.g., Wilson, 2002) which postulates that the function of cognition is to guide action, and cognitive mechanisms such as perception and memory must be assessed in terms of their ultimate contribution to goal-directed behaviour. Alternatively, the “*palm, fist, open*” suppression task developed for the purpose of this experiment may simply be too different from the coarser arm movements (*touch, pick up*, etc.) involved in the primary following instructions task to generate mutual interference. Indeed, participants reported that to aid accurate enactment of the hand sequence, they often verbalised the gestures which does not require kinaesthetic encoding. The dual-task costs on memory for instructions in all interference conditions could therefore have arisen through disruption to the storage of the verbal content of the sequences.

To investigate this possibility, a new motor suppression task was developed for use in Experiment 2. This involved gross motor movements with the morphological features of arm extension and hand manipulation that were more similar to the actions involved in the primary following instructions task. As in Experiment 1, the key aim of this study was to isolate the components of working memory involved in following instructions. It was predicted that the action advantage at recall would be significantly reduced only under concurrent motor suppression but would remain intact under dual-task conditions involving backward counting and articulatory suppression.

## Experiment 2

### Method

#### Participants

In total, 16 right-handed volunteers (10 females) participated in the study in return for a small honorarium. The mean age of the sample was 23.19 (*SD* = 3.17, range: 18-29) years. All were native English speakers, had normal or corrected to normal vision and no history of neurological disorders. Participants were recruited via the MRC Cognition and Brain Sciences Unit volunteer panel using an online booking system and none had taken part in Experiment 1.

#### Materials

##### Primary and secondary tasks

**Figure 2.**
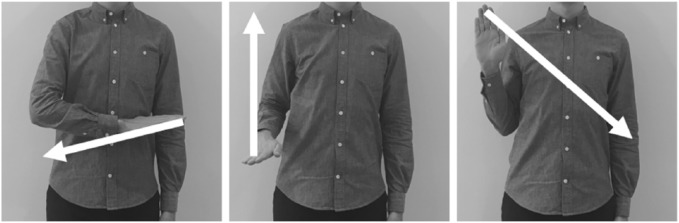
A schematic of the motor suppression task employed in Experiments 2 and 3. There were three gestures that flowed in a fluid sequence to form a movement. Each gesture can be described in terms of two features: onset location and movement. The sequence began with the participant’s right forearm in front of their chest pointing to the left (forearm horizontal to the floor). The forearm was then rotated externally with a horizontal movement to the right. The rotation ended when the fingertips (pointing forward) were approximately aligned to the participant’s elbow. The second gesture involved flexing the forearm upwards until it was parallel with the upper arm. For the third gesture, the forearm had to be rotated to the left and lowered in order to return to the original onset location.

The following instructions paradigm and the interference tasks were identical to those used in Experiment 1, with two exceptions. First, due to possible floor effects in the backward counting condition in Experiment 1, in this experiment, participants were asked to count aloud in decrements of two from an even two-digit number (e.g., *84, 82, 80*). Second, the motor suppression task involved three gross motor gestures (illustrated and described in Figure 2). The transitions between the gestures were fluid so that each movement ended in the onset location of the next gesture. The shapes of the right forearm and the right hand were held constant throughout (i.e., wrist straight and fingers touching). Like in Experiment 1, there were 12 trials within each block (two practice trials and 10 test trials).

#### Design and procedure

A 2 × 4 repeated measures design manipulating recall format (verbal, action) and concurrent interference (no interference, articulatory suppression, backward counting, motor suppression) were used. Each of the eight resulting conditions was performed in a separate block. Condition order was counterbalanced across participants, with all those of a particular recall format performed together. The order of the concurrent activities was randomised between participants.

Many of the procedural details were the same as for Experiment 1. However, unlike the previous experiment, in which the speed of the concurrent tasks was self-paced, a metronome fixed at a rate of 80 beeps per minute was used to pace performance of all secondary activities.

### Results

**Figure 3.**
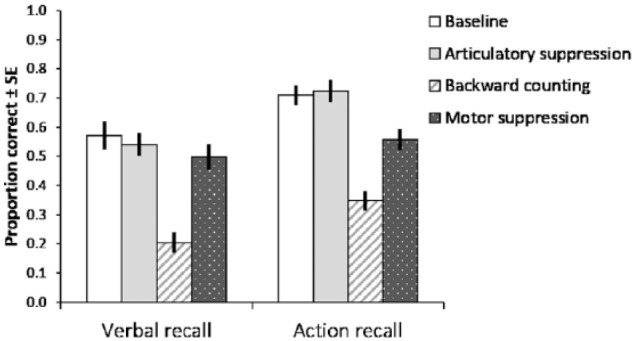
Mean proportion of action phrases correctly recalled in Experiment 2, as influenced by the three concurrent activities. Error bars represent standard error.

Figure 3 displays the accuracy scores across different experimental conditions of the following instructions task. As in Experiment 1, the dependant variable was the proportion of object–action pairs recalled in correct serial order. The data were submitted to a 2 × 4 ANOVA in which the type of recall (verbal, action) and secondary task (no interference, articulatory suppression, backward counting, motor suppression) were within-subjects factors. This revealed a significant main effect of concurrent task, *F*(3, 45) = 56.314, *MSE* = .025, *p* < .001, $\eta_{p}^{2}=.790$ (Mauchly’s test indicated that the assumption of sphericity had been violated, χ^2^(5) = 12.99; therefore, Greenhouse–Geisser–corrected statistics are reported, ε = .65). Simple main effects analysis revealed that performance in the baseline condition (aggregated across both types of recall) was significantly better than performance under motor suppression or in the presence of backward counting (both *p* < .001, all *p* values have been interpreted using the Bonferroni correction for multiple comparisons). There was, however, no difference in memory for instructions between the baseline and articulatory suppression conditions (*p* = 1.00). There was also a main effect of recall type, *F*(1, 15) = 17.127, *MSE* = .138, *p* = .001, $\eta_{p}^{2}=.533$, with better performance under conditions of enacted than verbal recall.

The concurrent task by recall type interaction term was also significant, *F*(3, 45) = 3.226, *MSE* = .026, *p* = .031, $\eta_{p}^{2}=.177$. Planned pairwise comparisons revealed that the action recall advantage was present in three of the four concurrent task conditions: with no concurrent task, *t*(15) = 3.102, *p* = .007, *d* = .78; with articulatory suppression, *t*(15) = 3.972, *p* = .001, *d* = .99; and also in the backward counting condition, *t*(15) = 4.053, *p* = .001, *d* = 1.01. In the motor suppression condition, the action recall advantage was not significant (*t*(15) = 1.584, *p* = .134, *d* = .40).

### Discussion

In this experiment, motor suppression selectively diminished the action advantage at recall. As in Experiment 1, the mnemonic benefit of action-based recall persisted in the presence of concurrent articulatory suppression and backward counting (see also Allen & Waterman, 2015; Yang et al., 2016; Yang et al., 2014). The negative impact of motor suppression on the action advantage is consistent with the hypothesis that the action advantage arises from the formation of temporary motoric representations of planned action sequences that can be disrupted by concurrent configural movements (i.e., movements in which the pattern or configuration of the body parts themselves is the goal for the action). This supports the idea that there may be a cognitive system dedicated to the short-term maintenance of movement trajectories and kinaesthetic representations (Smyth & Pendleton, 1989, 1990)—a conclusion which will be discussed further in the general discussion.

Concurrent articulatory suppression failed to disrupt memory for spoken instructions, which is at odds with reports from Yang et al. (2016) and Yang et al. (2014) and with the results of Experiment 1. This discrepancy could be attributed to the difficulty (i.e., speed of vocalisations) of the concurrent task which was set at a rate of 80 beeps per minute in the current experiment and self-paced in Experiment 1. The cognitive load imposed by articulating 80 words per minute may not have been sufficiently challenging to interfere with the encoding and retention of verbal instructions for all participants. In an attempt to rectify this inconsistency, the original self-paced articulatory suppression task used in Experiment 1 was employed in the next experiment.

The primary aim of Experiment 3 was to replicate the motor suppression effect observed in Experiment 2 that performance of gross motor gestures during the instruction task would diminish the action advantage at recall. The articulatory suppression task was changed from a fixed rate to self-paced, as in Experiment 1, with the aim of equating the demands across different participants.

## Experiment 3

### Method

#### Participants

In total, 16 right-handed volunteers (eight females) participated in the study in exchange for a small monetary reimbursement. The mean age of the sample was 23.25 (*SD* = 2.91) years ranging between 19 and 27 years. All were native English speakers, had normal or corrected to normal vision, and no history of neurological disorders. Participants were recruited via the MRC Cognition and Brain Sciences Unit volunteer panel using an online booking system and none had taken part in either Experiment 1 or 2.

#### Materials

The articulatory suppression task was identical to that used in Experiment 1, and the motor suppression task was the same as that used in Experiment 2. Task difficulty in the dual-task conditions (i.e., speed of performance) was set at an individual level. Participants were instructed to perform the concurrent activity continuously and consistently throughout and to be as fast and as accurate as possible.

#### Design and procedure

The experiment implemented a 2 × 3 repeated measures design manipulating concurrent interference (no interference, articulatory suppression, motor suppression) as a function of recall format (verbal, action). Each of the six resulting conditions was performed in a separate block. Condition order was counterbalanced across participants, with all those of a particular recall format performed together. The order of the interference conditions was randomised between participants. There were 12 trials within each block (two practice trials and 10 test trials). The procedure was identical to Experiment 1, except the backward counting task was not administered.

### Results

**Figure 4.**
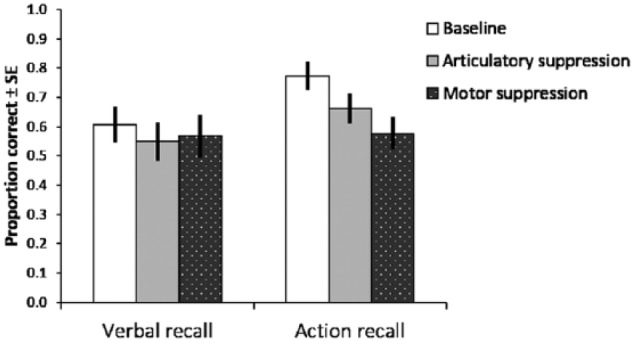
The effects of articulatory and motor suppression on performance accuracy in Experiment 3. Error bars denote standard error of the mean.

Accuracy data from the following instructions task is presented in Figure 4. A 3 (concurrent task) × 2 (recall type) repeated measures ANOVA revealed a significant main effect of recall type, *F*(1, 15) = 7.344, *MSE* = .030, *p* = .016, $\eta_{p}^{2}=.329$, with superior performance in the action recall conditions. There was also a significant main effect of concurrent task, *F*(2, 30) = 22.999, *MSE* = .005, *p* < .001, $\eta_{p}^{2}=.605$, reflecting the lower levels of performance under dual-task conditions. Simple main effects analysis revealed that performance under both concurrent tasks (aggregated across both types of recall) was significantly poorer than baseline (both *p* < .001, Bonferroni-adjusted). The interaction term between the two factors was also significant, *F*(2, 30) = 7.255, *MSE* = .028, *p* = .003, $\eta_{p}^{2}=.326$. Planned pairwise comparisons showed that action recall was significantly better than verbal recall in the baseline condition, *t*(15) = 3.931, *p* = .001, *d* = .98, and under concurrent articulatory suppression, *t*(15) = 3.331, *p* = .005, *d* = .83. However, there was no significant difference in performance between the verbal and action recall conditions under motor suppression (*t*(15) = .186, *p* = .855, *d* = .05).

### Discussion

The elimination of the action advantage following motor suppression involving arm movements that were observed in Experiment 2 was replicated in the present experiment. This concurrent activity impaired action but not verbal recall. This finding is consistent with our speculation that the action advantage is driven by the formation and maintenance of temporary kinaesthetic representations of planned action steps.

Experiment 3 also tested whether the diminished impact of articulatory suppression on the recall of spoken instructions seen in Experiment 2 relative to Experiment 1 was due to pacing the vocalisations with a metronome. There was evidence for this: as in Experiment 1, verbal recall was disrupted by concurrent articulatory suppression. We therefore conclude that the role played by the phonological store in the verbal recall of spoken instruction sequences, at least with this specific version of the paradigm, is substantial.

## General discussion

In three experiments, we tested the hypothesis that the ability to follow instructions depends in part on a limited-capacity store dedicated to the temporary retention of the motoric, spatial, and temporal features of intended actions. The new finding, replicated across two experiments, was that the advantage afforded to the recall of instructions by action over verbal repetition (action advantage) is disrupted by concurrent gross motor movements. However, it was undiminished by secondary tasks designed to disrupt the phonological or central executive components of working memory or by a concurrent fine motor movement task. These data are consistent with previous findings demonstrating that the action advantage is not driven by verbal or executive aspects of working memory (Yang et al., 2016; Yang et al., 2014) and provide broad support for Smyth and Pendleton’s (1989, 1990) proposal that a motor buffer is available to support the temporary maintenance of movement trajectories and kinaesthetic representations. The action advantage was not, however, disrupted by more precise sequences of manual movements. Because this sequence was difficult to produce at speed and easy to verbalise (i.e., *palm, fist, point*), it may have not have been guided by motoric representation in the motor stored but instead on verbal sequences held in the phonological loop. Alternatively, the precision required by the two sets of motor sequences—the fine-grained manual movements and the more general limb movements involved in the instructions task—may simply have been too distinct to generate high levels of interference.

The current findings suggest that working memory may depend in part on the cognitive systems that mediate body movements. Corresponding claims have been made on the basis of neuroimaging studies identifying functional links between linguistic and action-semantic systems of the human brain (e.g., Pulvermüller, 2001, 2005; Pulvermüller & Fadiga, 2010). It has been established that in situations in which complex motor patterns that are part of the semantic networks of the action words (e.g., kicking, jumping) are incompatible with the movement sequences executed, the respective motor circuits may compete with each other, possibly due to local cortical inhibition (for a formal model, refer to Garagnani & Pulvermüller, 2011; Garagnani, Wennekers, & Pulvermüller, 2008). This type of interference could have occurred in the current motor suppression condition. For example, if a participant was lifting her arm while simultaneously processing the word touch, there may have been interference between the semantic networks associated with the word touch and activation in the sensorimotor cortex evoked through the action of raising an arm. In essence, the current findings show that engaging the motor system can degrade working memory for action phrases. This, in turn, supports the idea that the sensorimotor system shares processing resources with these verbal working memory processes which is in line with a recent report from Shebani and Pulvermüller (2013) who concluded that body movements and working memory for action-related words share processing resources.

Across all three experiments, memory for instructions was impaired by concurrent articulatory suppression and backward counting, suggesting a significant role for both the phonological loop and central executive in instruction following. This is consistent with previous findings (Gathercole et al., 2008; Jaroslawska, Gathercole, Logie, & Holmes, 2016; Yang et al., 2016; Yang et al., 2014) and indicates that the crucial constraint when following practical instructions is not simply the passive storage of verbal information but rather the formation, maintenance, and accessibility of the representation of the required action sequence in working memory during the course of the performed action.

One possibility that has not yet been tested in the present series of experiments is that the action advantage is mediated by the visuospatial sketchpad component of Baddeley’s model of working memory. In the current paradigm, the visual display of the objects was in sight at all times to provide participants with the opportunity to utilise visuospatial cues in the environment in all experimental conditions. As a result, any task that required visual processing may have confounded performance by directing visual attention away from the cues in the display. In addition, recent studies suggest that there is a minimal contribution of the visuospatial sketchpad to instruction following (Jaroslawska, Gathercole, Logie, & Holmes, 2016), and that concurrent visuospatial sketchpad tasks, such as block tapping, have no impact on the action advantage (Yang et al., 2016). Moreover, Yang et al. (2016) reported that although blocking access to the visual display during encoding had a detrimental effect on overall performance, it failed to diminish the advantage afforded to physical action at recall. A further experiment including concurrent tasks taxing all hypothesised components of working memory would provide stronger evidence for the existence of a distinct motor store. As it is possible that the motor task used here may also have tapped memory for spatial information, it would be particularly valuable to assess the differential contributions of visuospatial and motoric representations to performing actions to command and to the action advantage more specifically.

To conclude, the data reported here support the idea that there may be a cognitive system dedicated to the temporary maintenance of spatial–motoric representations and that this store is the source of the action at recall advantage. This work has enhanced our understanding of the cognitive processes underlying the ability to adhere to instructions and it has generated important outcomes for the multi-component theory of working memory, pointing to a revised framework that includes a motor store. The evidence presented here provides some support for the amended model put forward by Baddeley et al. (2011) in that the storage of kinaesthetic information ought to be incorporated into the framework. Its precise relation to the visuospatial sketchpad and other aspects of working memory is yet to be explored, although the data collected here suggest that it is functionally distinct from both the phonological loop and central executive.
